# Are Single Polymer Network Hydrogels with Chemical and Physical Cross-Links a Promising Dynamic Vibration Absorber Material? A Simulation Model Inquiry †

**DOI:** 10.3390/ma13225127

**Published:** 2020-11-13

**Authors:** Leif Kari

**Affiliations:** The Marcus Wallenberg Laboratory for Sound and Vibration Research (MWL), Department of Engineering Mechanics, KTH Royal Institute of Technology, 100 44 Stockholm, Sweden; leifkari@kth.se; Tel.: +46-70-798-7974

**Keywords:** single polymer network hydrogel, chemical cross-link, physical cross-link, high loss factor, adhesion–deadhesion activity, dynamic vibration absorber, primary vibration system, simulation model, vibration reduction, smooth frequency dependence

## Abstract

Tough, doubly cross-linked, single polymer network hydrogels with both chemical and physical cross-links display a high loss factor of the shear modulus over a broad frequency range. Physically, the high loss factor is resulting from the intensive adhesion–deadhesion activities of the physical cross-links. A high loss factor is frequently required by the optimization processes for optimal performance of a primary vibration system while adopting a dynamic vibration absorber, in particular while selecting a larger dynamic vibration absorber mass in order to avoid an excess displacement amplitude of the dynamic vibration absorber springs. The novel idea in this paper is to apply this tough polymer hydrogel as a dynamic vibration absorber spring material. To this end, a simulation model is developed while including a suitable constitutive viscoelastic material model for doubly cross-linked, single polymer network polyvinyl alcohol hydrogels with both chemical and physical cross-links. It is shown that the studied dynamic vibration absorber significantly reduces the vibrations of the primary vibration system while displaying a smooth frequency dependence over a broad frequency range, thus showing a distinguished potential for the tough hydrogels to serve as a trial material in the dynamic vibration absorbers in addition to their normal usage in tissue engineering.

## 1. Introduction

Dynamic vibration absorbers are generally a simple and cost effective vibration reduction measure and consist in their simplest form of a mass and spring with natural frequency adjusted to result in a counter force at a desired frequency to reduce the vibrations of the primary vibration system they are attached to. Their application area is found in a wide range since their first patent by Frahm [1], including earthquake protection of engineering constructions, reduction of human walk induced vibrations of engineering constructions, reduction of wind induced power transmission line vibrations, reduction of driveline vibrations in vehicles and reduction of aircraft and spacecraft vibrations [2,3,4,5,6,7,8,9,10]. Over the years, the design of dynamic vibration absorbers has evolved in various directions, including multiple, nonlinear, composite, continuous, active and semi-active dynamic vibration absorbers, see, e.g., Refs. [3,4,5,6,8,11,12]. Commonly, damping is added to the dynamic vibration absorbers to increase their effective bandwidth, to dissipate mechanical energy and to reduce their sensitivity to design parameter deviations. However, the optimal loss factor as given by the optimization procedures [2,3,13,14] is frequently rather high, in particular while selecting a not too small dynamic vibration absorber mass to avoid an excess displacement amplitude of the dynamic vibration absorber spring. Nonetheless, the high loss factor may be difficult to obtain for conventional solid structural engineering materials, e.g., natural rubber. However, tough hydrogels that are frequently applied in tissue engineering, contain various cross-linked network configurations of polymer macromolecules and are heavily swollen with water, may also be an interesting vibration absorber spring material. Tough hydrogels have previously shown experimentally to be a possible vibration isolator material [15].

In this paper, tough, doubly cross-linked, single polymer network hydrogels simultaneously containing both chemical and physical cross-links [16,17,18,19,20,21,22,23,24,25,26,27,28,29,30,31,32,33,34,35,36,37,38,39] are theoretically examined as a plausible dynamic vibration absorber spring material. The study is an extension of the preliminary investigation of vibration isolators published at the Medyna 2020 conference [40] to dynamic vibration absorbers. A special focus is on the dually cross-linked polyvinyl alcohol hydrogels where stress–strain models exist, see, e.g., Refs. [19,22,23,25,37,39,41]. The stress–strain models for dually cross-linked polyvinyl alcohol hydrogels include fractional time power dependence and finite deformations [19], survivability functions using several material parameters and finite deformations [23,25,37,41], generalized Stokes–Einstein equation [22] and fractional derivatives [39]. The fractional derivative model is suitable while only using 4 material parameters, displays an associative Rouse mode behavior [42] of the shear loss modulus in the low-frequency range with a frequency dependence of fractional order 1/2, applies physically intelligible material parameters, displays an additive split of the contributions from the chemical and physical cross-links and fits experimental results excellently [39]. Fractional derivative models are frequently used in visco-elasticity, see, e.g., Refs. [43,44,45,46,47,48,49,50,51,52,53,54,55,56,57,58,59,60,61,62,63,64,65,66,67,68,69,70,71,72,73,74,75,76,77,78,79,80,81,82,83,84,85,86,87,88,89,90,91], are used in modelling chemical and physical ageing of rubber [92,93] and in other topics, as presented in Machado et al. [94] and Rossikhin and Shitikova [95]. Doubly cross-linked, single polymer network hydrogels generally display a tunable frequency for maximum high loss modulus and a tunable low and high frequency shear modulus [31], thus making them suitable as a trial material in dynamic vibration absorber springs in addition to their normal usage in tissue engineering.

A model for the response of a primary vibration system and that of the dynamic vibration absorber is developed while using the newly developed stress–strain model for the doubly cross-linked, single network polyvinyl alcohol hydrogels [39]. The possibility of using tough hydrogel material in dynamic vibration absorber springs is theoretically investigated in this study by a simulation model, with special focus on the vibration reducing capacity of the dynamic vibration absorber.

## 2. Materials and Methods

**Figure 1:**
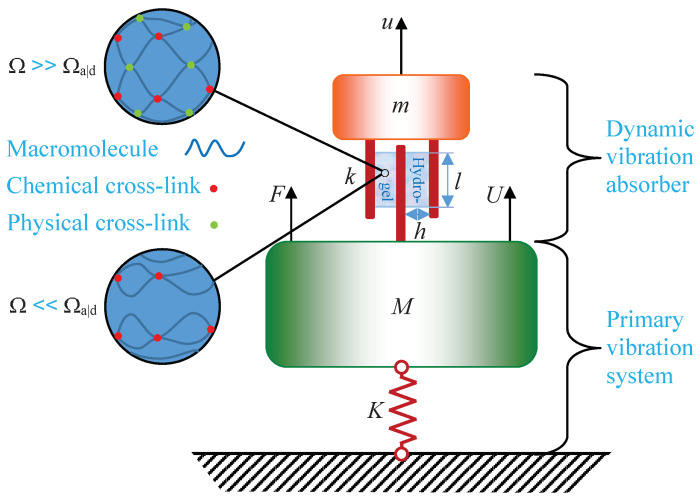
A primary vibration system attached to a dynamic vibration absorber consisting of tough, single polymer network hydrogel blocks in a simple shear configuration with both chemical and physical cross-links.

Consider the primary vibration system in Figure 1, consisting of a rigid body of mass *M* and an idealized spring of stiffness *K* attached to a rigid foundation, displaying a natural frequency

(1) $$\Omega_{0}=\sqrt{\frac{K}{M}},$$

without the attached dynamic vibration absorber. A dynamic vibration absorber consisting of a rigid body of mass *m* and a spring composed of two doubly cross-linked, single network hydrogel rectangular blocks of dimension h×l×w in a simple shear configuration, with the total stiffness

(2) $$k=2\frac{lw\mu }{h},$$

is connected to the primary vibration system, where the shear modulus

(3) $$\mu =\mu_{\mathrm{st}}\left\{1+C\sqrt{i\frac{\Omega }{\Omega_{a|d}}}+\frac{△\sqrt{i\frac{\Omega }{\Omega_{a|d}}}}{1+\sqrt{i\frac{\Omega }{\Omega_{a|d}}}}\right\},$$

according to the 4-parameters model in Kari [39], i is the imaginary unit, $\mu_{\mathrm{st}}$ is the static shear modulus, $\Omega_{a|d}$ is the angular frequency for maximum loss modulus (assuming C≈0), △ is a non-dimensional relaxation intensity, C is the chemical Rouse stress intensity factor and Ω is the angular excitation frequency. The equations of motion read

(4) $$-M\;\hat{U}\Omega^{2}+K\hat{U}+k\left(\hat{U}-\hat{u}\right)={\left(\frac{\Omega }{\Omega_{0}}\right)}^{\alpha }\hat{F}$$

and

(5) $$-m\;\hat{u}\;\Omega^{2}+k\left(\hat{u}-\hat{U}\right)=0,$$

respectively, where the mass displacements

(6) $$U=ℜ\{\hat{U}e^{i\;\Omega \;t}\}$$

and

(7) $$u=ℜ\{\hat{u}e^{i\;\Omega \;t}\},$$

respectively, the excitation force

(8) $$F={\left(\frac{\Omega }{\Omega_{0}}\right)}^{\alpha }ℜ\left\{\hat{F}e^{i\;\Omega \;t}\right\},$$

*t* is the time, $\hat{\left(•\right)}$ is the peak value of (•) (generally complex valued), *ℜ* is the real part and −2≤α≤2, generally, is the frequency dependence of the excitation force with α=0 approximating a flat force spectrum and α=2 a force spectrum similar to that of an unbalanced rotating mass. The resulting normalized mass displacements read

(9) $$\frac{\hat{U}}{U_{\mathrm{st}}}=f^{\alpha }\frac{f_{0}^{2}\left\{1+C\sqrt{i\frac{f}{f_{a|d}}}+\frac{△\sqrt{i\frac{f}{f_{a|d}}}}{1+\sqrt{i\frac{f}{f_{a|d}}}}\right\}-f^{2}}{f_{0}^{2}\left\{1+C\sqrt{i\frac{f}{f_{a|d}}}+\frac{△\sqrt{i\frac{f}{f_{a|d}}}}{1+\sqrt{i\frac{f}{f_{a|d}}}}\right\}\left(1-f^{2}\left[1+\chi \right]\right)\;+\;f^{2}\left(f^{2}-1\right)}$$

and

(10) $$\frac{\hat{u}}{U_{\mathrm{st}}}=f^{\alpha }f_{0}^{2}\frac{1+C\sqrt{i\frac{f}{f_{a|d}}}+\frac{△\sqrt{i\frac{f}{f_{a|d}}}}{1+\sqrt{i\frac{f}{f_{a|d}}}}}{f_{0}^{2}\left\{1+C\sqrt{i\frac{f}{f_{a|d}}}+\frac{△\sqrt{i\frac{f}{f_{a|d}}}}{1+\sqrt{i\frac{f}{f_{a|d}}}}\right\}\left(1-f^{2}\left[1+\chi \right]\right)\;+\;f^{2}\left(f^{2}-1\right)},$$

respectively, where the normalized excitation frequency

(11) $$f=\frac{\Omega }{\Omega_{0}},$$

the normalized natural frequency for dynamic vibration absorber

(12) $$f_{0}=\frac{1}{\Omega_{0}}\sqrt{\frac{k_{\mathrm{st}}}{m}},$$

without damping and with its spring attached to a rigid foundation, the normalized frequency for maximum loss modulus

(13) $$f_{a|d}=\frac{\Omega_{a|d}}{\Omega_{0}},$$

the total static stiffness

(14) $$k_{\mathrm{st}}=2\frac{lw\mu_{\mathrm{st}}}{h},$$

the mass ratio

(15) $$\chi =\frac{m}{M}$$

and the static displacement of the primary vibration system and of the dynamic vibration absorber mass

(16) $$U_{\mathrm{st}}=\frac{\hat{F}}{K},$$

at vanishing normalized frequency, at no gravity and with α=0. The total stiffness (2) is valid as long as the (half) shear wavelength is much longer than the height *h* of the rectangular hydrogel block—that is, as long as the Helmholtz’ number

(17) $$He=h\Omega \sqrt{\frac{\rho }{\left|\mu \right|}}\ll \pi ,$$

where ρ is the hydrogel density.

## 3. Results and Discussion

### 3.1. Material and Dynamic Vibration Absorber Parameters

The normalized natural frequency for the dynamic vibration absorber $f_{0}$ (12), at a given natural frequency $\Omega_{0}$ for the primary system, is tunable by selecting an appropriate mass *m*, an appropriate chemical cross-link density [31] to modify the static modulus $\mu_{\mathrm{st}}$ together with an appropriate hydrogel block dimension h×l×w to jointly modify the total static stiffness (14) for the dynamic vibration absorber. The relaxation intensity △ is tunable by selecting an appropriate physical-to-chemical cross-link density at the full physical cross-link activity [31]. The normalized frequency for maximum loss modulus $f_{a|d}$ is tunable by selecting an appropriate metal ion to modify the kinetics and thermodynamics of the adhesion–deadhesion activity of the physical cross-links [31]. It should be noted that polyvinyl alcohol hydrogels have a unique class of physical cross-links, namely borate esterification, thus partially limiting the variation of the kinetics and thermodynamics of the adhesion–deadhesion activity of the physical cross-links [31]. Nevertheless, the study undertaken in this paper is relevant for all doubly cross-linked, single polymer network hydrogels with both chemical and physical cross-links where the stress–strains relation (3) is relevant, and in particular interesting for those hydrogels with a greater variation capacity of the physical cross-links. Finally, the chemical Rouse stress intensity factor C is normally very small, modeling the slight increase with the normalized frequency of the chemical cross-link contribution to the normalized storage modulus and the chemical cross-link contribution to the normalized loss modulus [39] found in measurements while normally being much smaller than the corresponding contribution from the physical cross-links [19,35].

### 3.2. Shear Modulus

**Figure 2:**
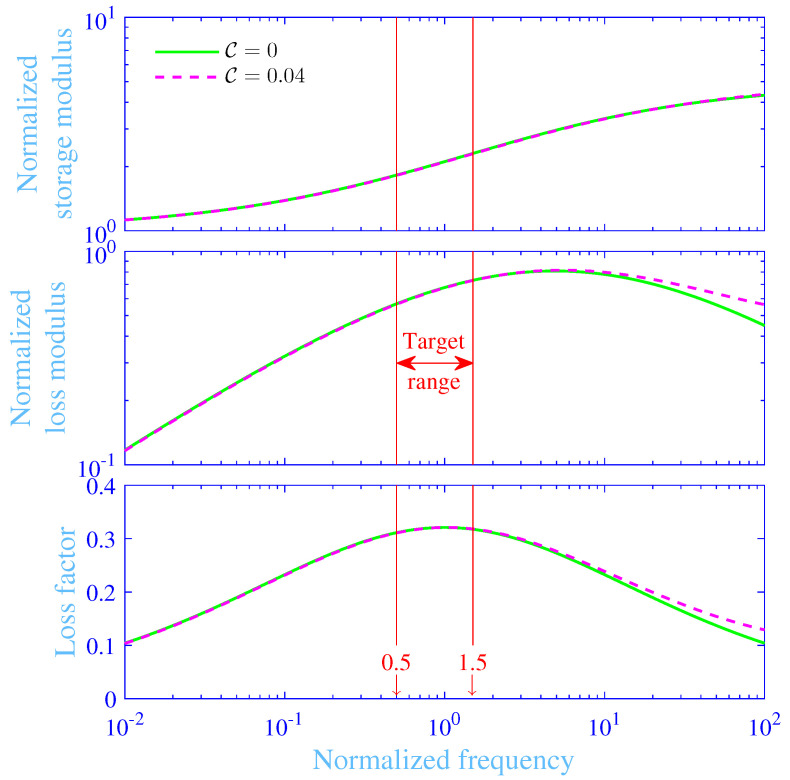
Normalized storage modulus, normalized loss modulus and loss factor versus the normalized frequency for the studied single network hydrogel at the chemical Rouse stress intensity factors C=0 and 0.04, with △=3.9055 and $f_{a|d}=4.9055$.

The normalized storage modulus $ℜ\left(\mu \right)/\mu_{\mathrm{st}}$, normalized loss modulus $ℑ\left(\mu \right)/\mu_{\mathrm{st}}$ and loss factor η=ℑ(μ)/ℜ(μ) versus the normalized frequency *f* are in Figure 2, with the relaxation intensity △=3.9055 and the normalized frequency for the maximum normalized loss modulus $f_{a|d}=4.9055$ (assuming C≈0), where *ℑ* denotes imaginary part. The material parameter values adopted will be further explained in Section 3.3. The chemical Rouse stress intensity factor is set to zero C=0 for the green solid line (**—**) and to the average of the hydrogels studied in Kari [39]—that is C=0.04, for the magenta dashed line (**- - -**), the former corresponding to the 3-parameter model while the latter to the 4-parameter model in Kari [39]. Clearly, the curves with and without the chemical Rouse stress intensity factor almost match each other; the only discrepancies are at the high normalized frequency regions of the normalized loss modulus and of the loss factor where the curves including the chemical Rouse stress intensity factor display slightly higher values. This is also true for the normalized storage modulus although the dissimilarity is exceptionally small and may be perceived as ocularly identical. Moreover, the normalized storage modulus shows a comparatively small value in the low normalized frequency range due to that merely only the chemical cross-links are active, goes trough a transition region around f=1, ascends with the increasing normalized frequency and is ending at a high value at the high normalized frequency range where both the chemical and physical cross-links are fully active [19,21,31,39]. Similarly, the normalized loss modulus shows a small value in the low normalized frequency range, increases with the normalized frequency, peaks at $f\approx f_{a|d}$ and decreases with the normalized frequency in the higher normalized frequency region. Finally, the loss factor shows a small value in the low normalized frequency range, increases with the normalized frequency, peaks at f≈1 and decreases with normalized frequency in the higher normalized frequency region.

It is straightforward to derive, from the shear modulus model (3) and Equation (13), that the maximum loss factor $\eta_{\max}=△/\left({\left[\sqrt{2}+\sqrt{1+△}\;\right]}^{2}-1\right)$ at the normalized frequency $f_{\max}\;=\;f_{a|d}/\left(1\;+\;△\right)$ for C=0, here reading $\eta_{\max}=32.1$% and $f_{\max}=1.00$, respectively. Furthermore, $\eta_{\max}=32.1$% and $f_{\max}=1.03$, respectively, for C=0.04, thus being approximately identical to those of hydrogels without the chemical Rouse stress intensity factor. A target normalized frequency range for the dynamic vibration absorber is depicted in Figure 2 and explained in the next Section 3.3.

### 3.3. Optimization

The maximum loss factor in Figure 2 is relatively high ($\eta_{\max}=32.1$%) and may be difficult to obtain for conventional solid structural engineering materials, e.g., for natural rubber. Nevertheless, a high loss factor is required for an optimal performance [2,3,13,14], in particular for high mass ratios χ. The high mass ratios are needed to avoid an excess displacement amplitude of the dynamic vibration absorber springs. The main idea in this paper is to use the tough, doubly cross-linked, single network hydrogel as the spring material in the dynamic vibration absorber and to tune its loss factor η to peak at f=1—that is at the normalized natural frequency for the primary vibration system without the attached dynamic vibration absorber. Consequently, the dynamic vibration absorber is set to obey $f_{a|d}/\left(1+△\right)=1$. The optimization process is considered next.

**Figure 3:**
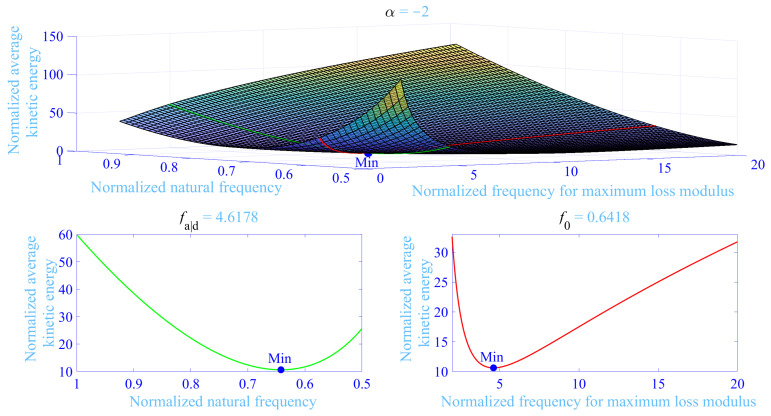
Frequency averaged normalized kinetic energy of the primary vibration system *M* versus the normalized natural frequency for dynamic vibration absorber and versus the normalized frequency for maximum loss modulus, with C=0.04 and χ=1/10. The global minimum of the frequency averaged maximum normalized kinetic energy [Min] is shown with a blue bullet (•) being located at the crossing between the green and red curves.

The maximum kinetic energy of the primary vibration system normalized with its spring energy at the maximum displacement is

(18) $$W_{\mathrm{kin}}=\frac{M(|\hat{U}{\left|\Omega \right)}^{2}}{2}\frac{2}{K\;|U_{\mathrm{st}}{|}^{2}},$$

reading

(19) $$W_{\mathrm{kin}}={\left|\frac{\hat{U}}{U_{\mathrm{st}}}\right|}^{2}f^{2},$$

with $\hat{U}/U_{\mathrm{st}}$ given in Equation (9). The frequency averaged maximum normalized kinetic energy of the primary vibration system

(20) $${\bar{W}}_{\mathrm{kin}}=\frac{1}{f_{+}-f_{-}}\int_{f_{-}}^{f_{+}}{\left|\frac{\hat{U}}{U_{\mathrm{st}}}\right|}^{2}f^{2}df,$$

where $f_{-}<1<f_{+}$ are the normalized frequency limits. The target normalized frequency range depicted in Figure 2 applies $f_{-}=0.5$ and $f_{+}=1.5$ with f=1 being their average value, thus well including the normalized natural frequency for the primary vibration system and the extra resonance peaks due to the application of the dynamic vibration absorber while in addition forcing the loss factor of the hydrogel in the attached dynamic vibration absorber to peak at the same normalized frequency by matching $f_{a|d}$ to be identical to 1+△. The resulting optimized hydrogel material and dynamic vibration absorber parameters ($f_{0},△,f_{a|d}↔1+△$) for α=−2,0,+2 and χ=1/5,1/10,1/20,1/40 are given in Table 1 and Table 2 in green for C=0 and C=0.04, respectively, while minimizing the frequency averaged maximum normalized kinetic energy of the primary vibration system in Equation (20) $\left[\min\left({\bar{W}}_{\mathrm{kin}}\right)\right]$, with $f_{-}=0.5$ and $f_{+}=1.5$ and using the constrained non-linear multi-variable programming solver fmincon from Matlab${}^{®}$. Clearly, the parameter results for C=0 and C=0.04 are similar, displaying an increase of △ and $f_{a|d}$, with increasing mass ratio χ and with increasing α. Furthermore, $f_{0}$ increases with increasing α and decreases with increasing mass ratio χ. In conclusion, it is possible to meet the necessary high loss factor values for high mass ratios χ with doubly cross-linked, single network hydrogels by adjusting the maximum loss factor to peak at f=1 and to tune the stress intensity △ to meet the optimized values in Table 1 and Table 2. In passing, it should be noted that it is possible to meet the required loss factor and the normalized natural frequency for the dynamic vibration absorber values by other combinations, for example, by higher stress intensities than the tabulated together with the loss factor peaks outside the target normalized frequency range for the dynamic vibration absorber. This is in particular true for the lower mass ratios χ where the demand for a high loss factor is absent. To further examine the resulting parameters found from the optimization process, a three-dimensional plot is in Figure 3 displaying the frequency averaged maximum normalized kinetic energy of the primary vibration system versus the normalized natural frequency for dynamic vibration absorber $0.5\leq f_{0}\leq 1$ and versus the normalized frequency for maximum loss modulus $2\leq f_{a|d}\leq 20$ for α=−2, χ=1/10 and C=0.04. Furthermore, two slices from the same three-dimensional plot are presented; the first is showing the frequency averaged maximum normalized kinetic energy of the primary vibration system versus the normalized natural frequency for dynamic vibration absorber $0.5\leq f_{0}\leq 1$ at the optimum value of the normalized frequency for maximum loss modulus $f_{a|d}^{\mathrm{opt}}=1+{△}^{\mathrm{opt}}=4.6178$ given from Table 2, while the second is showing the frequency averaged maximum normalized kinetic energy of the primary vibration system versus the normalized frequency for maximum loss modulus $2\leq f_{a|d}\leq 20$ at the optimum value of the normalized natural frequency for dynamic vibration absorber $f_{0}^{\mathrm{opt}}=0.6418$ given from the same Table 2. Clearly, the found minimum of the frequency averaged maximum normalized kinetic energy of the primary vibration system $\left[\min\left({\bar{W}}_{\mathrm{kin}}\right)\right]$ is definitely a global minimum at the parameter values given in Table 2.

**Figure 4:**
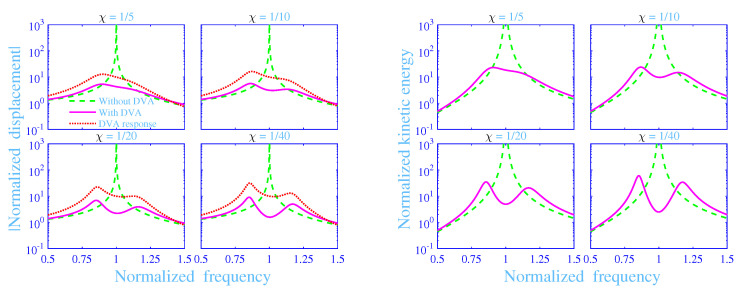
Absolute value of the normalized displacement and the normalized kinetic energy of the mass *M* versus the normalized frequency with and without the dynamic vibration absorber (DVA) together with the absolute value of the normalized displacement of the mass *m* versus the normalized frequency, at various mass ratios χ, with α=0 and C=0.04.

The absolute value of the normalized displacement $|\hat{U}/U_{\mathrm{st}}|$ and the normalized kinetic energy $W_{\mathrm{kin}}$ of mass *M* versus the normalized frequency *f* with and without the dynamic vibration absorber are in magenta solid lines (**—**) (with) and in green dashed lines (**- - -**) (without) in Figure 4, for the mass ratios χ=1/5,1/10,1/20,1/40 with α=0 and C=0.04, while using the optimized parameter values in Table 2. Likewise, the absolute value of the normalized displacement $|\hat{u}/U_{\mathrm{st}}|$ of the mass *m* of the dynamic vibration absorber versus the normalized frequency *f* is in red dotted lines (•••) in the same Figure 4. Clearly, the absolute value of the normalized displacement and the normalized kinetic energy of the mass *M* without the dynamic vibration absorber show a sharp resonance peak at f=1—in theory $|\hat{U}/U_{\mathrm{st}}|\to \infty$ and $W_{\mathrm{kin}}\to \infty$ at f=1. Although it is possible to include some damping into the primary vibration system, the undamped system is nevertheless a sufficiently appropriate representation of the primary vibration system as dynamic vibration absorbers are generally applied to undamped and lightly damped primary vibration systems whereas considerably more damped systems calls for other vibration reducing measures including structural modifications. Obviously, the dynamic vibration absorber significantly reduces the absolute value of the normalized displacement and the normalized kinetic energy of the mass *M*, displaying a smooth normalized frequency dependence over the normalized frequency region considered. Their fluctuations increases slightly with decreased mass ratio χ over the normalized frequency region considered. The absolute value of the normalized displacement of the mass *m* displays similar behavior as the absolute value of the normalized displacement of the mass *M* with the dynamic vibration absorber although its value is significantly higher than for the mass *M* in a normalized frequency region close to f=1, say 0.75≤f≤1.25. The smaller the mass ratio χ, the higher is the absolute value of the normalized displacement of the mass *m* within the same normalized frequency region.

It should be noted that the optimization procedure applied in this paper minimizes the frequency averaged maximum normalized kinetic energy of the primary vibration system while other frequently applied optimization procedures minimize the normalized displacement of the primary vibration system [2,3,13], see discussion in Asami and Nishihara [14]. Furthermore, there are fixed-point handbook methods developed for viscously and hysterically damped dynamic vibration absorbers [2,3,13], where the normalized displacement of the primary vibration system at two specific points in the frequency response curve, shown to be independent on the damping applied, are forced by construction to have equal amplitudes by adjusting the normalized natural frequency for the dynamic vibration absorber, while their derivatives with respect to the normalized frequency are forced by construction to be zero by adjusting the damping. However, the target in this paper is not to minimize the normalized displacement of the primary vibration system, differing from the normalized kinetic energy of the primary vibration system in Equation (19) by a missing factor $f^{2}$ that is weighting the normalized displacement (squared) more at the higher frequencies. Moreover, the damping model successfully applied for the tough hydrogel used [39] is based on fractional derivatives with results that lie between those of viscous and hysteric damping. In conclusion, the optimization process applied in this paper is not likely to result in equal amplitudes of the normalized displacements of the primary vibration system at the two peaks nor to exactly the same amplitudes of the two peaks of the normalized kinetic energy of the primary vibration system as the procedure results in a global minimum of the frequency averaged normalized kinetic energy of the primary vibration system, see Figure 3.

**Figure 5:**
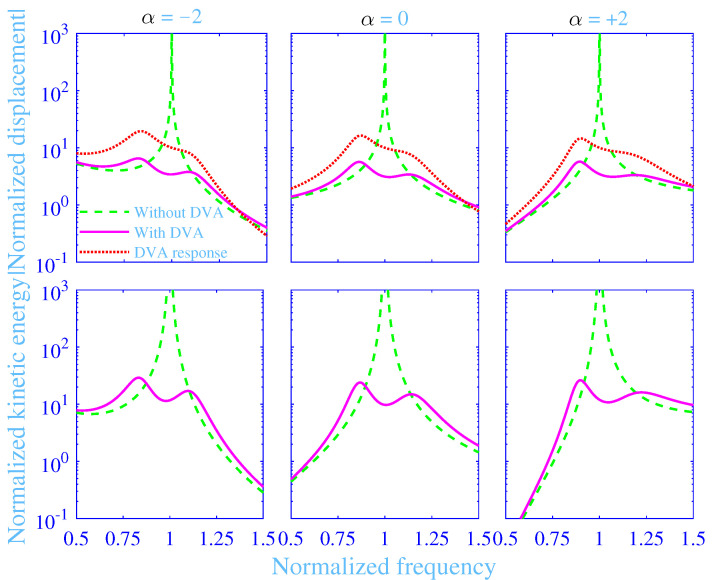
Absolute value of the normalized displacement and the normalized kinetic energy of the mass *M* versus the normalized frequency with and without the dynamic vibration absorber (DVA) together with the absolute value of the normalized displacement of the mass *m* versus the normalized frequency, at various normalized frequency dependencies of the excitation force α, with C=0.04 and χ=1/10.

The absolute value of the normalized displacement $|\hat{U}/U_{\mathrm{st}}|$ and the normalized kinetic energy $W_{\mathrm{kin}}$ of the mass *M* versus the normalized frequency *f* with and without the dynamic vibration absorber are in magenta solid lines (**—**) (with) and in green dashed lines (**- - -**) (without) in Figure 5, for the normalized frequency dependencies of the excitation force α=−2,0,+2 with C=0.04 and χ=1/10, while using the optimized parameter values in Table 2. Similarly, the absolute value of the normalized displacement $|\hat{u}/U_{\mathrm{st}}|$ of the mass *m* of the dynamic vibration absorber versus the normalized frequency *f* is in red dotted lines (•••) in the same Figure 5. Clearly, the curves display similar behavior as the corresponding curves in Figure 4 (α=0), however, with increased values in the low normalized frequency range and decreased values in the high normalized frequency range for α=−2 and the opposite—decreased values in the low normalized frequency range and increased values in the high normalized frequency range for α=+2. This is not surprising as the normalized displacements have a factorial α-dependence—that is, proportional to $f^{\alpha }$ and the normalized kinetic energy has a factorial (2α+2)-dependence—that is, is proportional to $f^{2(\alpha +1)}\;$, according to Equations (9), (10) and (19). In passing, it is noted that the two peaks for the normalized kinetic energy curves for α=−2 in Figure 5 display slightly different peak values. The reason is that the global optimization process shown in Figure 3 is minimizing the frequency averaged normalized kinetic energy and not just their peak values.

The resulting optimized hydrogel material and dynamic vibration absorber parameters ($f_{0},△,f_{a|d}$) for a full optimization with α=0, C=0.04 and χ=1/10 are given in Table 3 in green, while minimizing the frequency averaged maximum normalized kinetic energy of the primary vibration system Equation (20) $\left[\min\left({\bar{W}}_{\mathrm{kin}}\right)\right]$, with $f_{-}=0.5$ and $f_{+}=1.5$, using the same constrained non-linear multi-variable programming solver fmincon from Matlab${}^{®}$, however without constraining the normalized frequency of the loss factor peak $f_{\max}$ to coincide with f=1 (assuming C≈0)—that is, $f_{a|d}↮1+△$, normally. For comparison, the corresponding optimized hydrogel material and dynamic vibration absorber parameters ($f_{0},△,f_{a|d}↔1+△$) from Table 2 are given in the same Table 3 in green, with the constraint $f_{a|d}↔1+△$—that is, constraining the normalized frequency of the loss factor peak $f_{\max}$ to coincide with f=1 (assuming C≈0). Furthermore, a conventional solid structural engineering material acting as the dynamic vibration absorber spring material is compared to the tough, single network hydrogel material studied in this paper. To this end, the unfilled natural rubber (NR) standard Malaysian rubber (SMR) general purposes (GP), studied in Kari [52], is selected. The reader is referred to Kari [52] for the details over the ingredients, material process and the material property measurements. The shear modulus model (3) needs to be modified by setting C=0, △=276 and by transforming all $\surd if/f_{a|b}^{\;\;\neg }$ into ${\left(if/f_{a|d}\right)}^{0.657}\;$, with $\Omega_{a|d}=3.3987\;\times \;10^{8}\;$rad/s. The resulting optimized NR material parameters and dynamic vibration absorber parameter $f_{0}$ for $\Omega_{0}=1,10,100\;$rad/s with α=0, C=0, △=276 and χ=1/10, are given in Table 3 in green. Finally, the minimum frequency averaged maximum normalized kinetic energy of the primary vibration system Equation (20) $\left[\min\left({\bar{W}}_{\mathrm{kin}}\right)\right]$ is given in the same Table 3. Clearly, $\min[{\bar{W}}_{\mathrm{kin}}]$ is almost identical for the full optimization and the constrained optimization of the hydrogel dynamic vibration absorber with $f_{a|d}↔1+△$, displaying a difference at the third digit, corresponding to a 0.1% difference, while the corresponding minimum frequency averaged maximum normalized kinetic energy of the primary vibration system for the natural rubber dynamic vibration absorber shows considerably larger values,being 320,71.0 and 16.9 times larger than for the constrained optimization of the hydrogel dynamic vibration absorber, for $\Omega_{0}=1,\;10\;\;\mathrm{and}\;100\;$rad/s, respectively.

**Figure 6:**
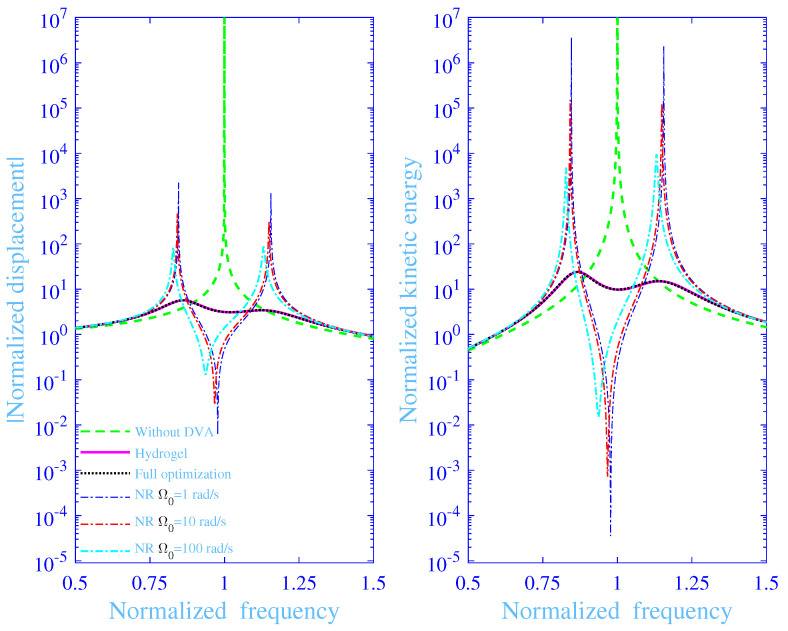
Absolute value of the normalized displacement and the normalized kinetic energy of the mass *M* versus the normalized frequency with and without the dynamic vibration absorber (DVA) together with the absolute value of the normalized displacement of the mass *m* versus the normalized frequency, for the hydrogel and the natural rubber dynamic vibration damping at various natural frequencies for the primary vibration system $\Omega_{0}$, with α=0 and χ=1/10.

The absolute value of the normalized displacement $|\hat{U}/U_{\mathrm{st}}|$ and the normalized kinetic energy $W_{\mathrm{kin}}$ of the mass *M* versus the normalized frequency *f*, with and without the dynamic vibration absorber, are in Figure 6 with α=0 and χ=1/10, while using the optimized parameter values in Table 3. The hydrogel dynamic vibration absorber results with the full optimization are in black dashed lines (**- - -**) and with $f_{a|d}↔1+△$ constraint in magenta solid lines (**—**). The natural rubber dynamic vibration absorber results are in blue dash–dotted lines (**– •**), red dash–dotted lines (**– •**) and in cyan dash–dotted lines (**– •**), for $\Omega_{0}=1,\;10\;\;\mathrm{and}\;100\;$rad/s, respectively. The curves without the dynamic vibration absorber are in green dashed lines (**- - -**) in the same Figure 6. Clearly, the hydrogel curves with and without the constraint $f_{a|d}↔1+△$ coincide—not surprisingly as their minimum frequency averaged maximum normalized kinetic energy of the primary vibration system Equation (20) $\left[\min\left({\bar{W}}_{\mathrm{kin}}\right)\right]$ nearly coincides in Table 3. Consequently, the design procedure to tune the normalized frequency $f_{\max}$ for the maximum loss factor to meet $f_{\max}=1$, by tuning the normalized frequency for the maximum normalized storage modulus $f_{a|d}$ to meet $f_{a|d}↔1+△$ results in a sufficiently low $\min({\bar{W}}_{\mathrm{kin}})$ (assuming C≈0), close to its global minimum (using full optimization), while in addition being a physically intelligible design process. Conversely, the natural rubber curves deviate significantly from the hydrogel curves—the former displaying two resonance peaks, below and above the normalized resonance peak frequency for the primary vibration system without the dynamic vibration absorber and one anti-resonance trough close to the normalized natural frequency for the primary vibration system without the dynamic vibration absorber. The resonance peak magnitudes are finite, displaying reduced peak magnitudes and reduced normalized peak frequencies with increasing $\Omega_{0}$—not surprising as the loss factor for the natural rubber increases within the normalized target frequency range, around f=1, with increasing $\Omega_{0}$. However, their loss factor is nevertheless very small, reading 0.059, 0.27 and 1.2% for $\Omega_{0}=1,\;10\;\;\mathrm{and}\;100\;$rad/s, respectively, and does not meet the high values as required by the optimization procedures [2,3,13,14]. Thus, this results in high peak resonance magnitudes and high values of the frequency averaged maximum normalized kinetic energy of the primary vibration system, the former being obvious in Figure 6 and the latter in Table 3. It is possible to select a higher damping rubber material than natural rubber, however generally to the cost of reduced mechanical properties, including lower strength, elasticity and wear resistance. Another high-damping possibilities include friction, sand and hydraulic damping components.

### 3.4. Chemical and Physical Cross-Links

The studied, doubly cross-linked, single network hydrogels contain simultaneously both chemical and physical cross-links where the chemical cross-links essentially contributes to the static elasticity while the physical cross-links to the frequency dependent loss modulus and to the frequency dependent part of the storage modulus. Physically, only the chemical cross-links are active at very low normalized frequencies f≪1 while the physical cross-links are essentially de-bonded due to the very long time frame of the mechanical oscillations resulting in a sufficiently long time for the physical cross-links to de-bond after bonding. The reader is referred to, e.g., Refs. [19,21,31,39] for more details about the physical interpretations. Briefly, the time frame for the mechanical oscillations is decreased at increased normalized frequency, resulting in more physical cross-links that are active—the higher the normalized frequency, the more physical cross-links are active generating both elasticity and losses, the latter due to the adhesion–deadhesion activities of the physical cross-links. The adhesion–deadhesion activities are at their maximum at a normalized frequency close to $f=f_{a|d}$, as is the normalized loss modulus while neglecting the small contribution from the chemical cross-links to the normalized loss modulus. The elasticity increases further at even higher normalized frequencies due to more contributions from the physical cross-links resulting in a pure and high elasticity at the high normalized frequency end, while neglecting the small contribution from the chemical cross-links to the normalized loss modulus. Although the normalized frequency for the maximum normalized loss modulus is $f_{a|d}$ (neglecting the small contribution from chemical cross-links), the normalized frequency for the maximum loss factor $f_{\max}$ is normally substantially smaller—namely $f_{\max}=f_{a|d}/\left(1+△\right)$ (also neglecting the small contribution from chemical cross-links). This is due to the definition of the loss factor, being the ratio between the loss modulus and the storage modulus, displaying a maximum at a normalized frequency smaller than the normalized frequency for the maximum normalized loss modulus since the storage modulus in the denominator increases continuously with increasing normalized frequency. An inclusion of the chemical Rouse stress intensity factor results in a small, square root normalized frequency dependent contribution to the normalized storage and to the normalized loss modulus, over the whole normalized frequency range within the considered normalized frequency range.

It is possible to additively split the shear modulus (3) into chemical and physical cross-link parts—$\mu^{*}=\mu_{\mathrm{Ch}}^{*}+\mu_{\mathrm{Ph}}^{*}$, where the normalized shear modulus $\mu^{*}=\mu /\mu_{\mathrm{st}}$, Ch denotes chemical and Ph physical cross-links, according to the model in Kari [39]. Inserting this additive split of the normalized shear modulus into the normalized mass displacements Equations (9) and (10) gives

(21) $$\begin{array}{cc} \frac{\hat{U}}{U_{\mathrm{st}}}=f^{\alpha } & \left\{f_{0}^{2}[\overset{\mu_{\mathrm{Ch}}^{*}}{\overset{︷}{\underset{ℜ\mu_{\mathrm{Ch}}^{*}}{\underset{︸}{1+C\sqrt{\frac{f}{2f_{a|d}}}}}+\;\;i\underset{ℑ\mu_{\mathrm{Ch}}^{*}}{\underset{︸}{C\sqrt{\frac{f}{2f_{a|d}}}}}}}+\overset{\mu_{\mathrm{Ph}}^{*}}{\overset{︷}{\underset{ℜ\mu_{\mathrm{Ph}}^{*}}{\underset{︸}{\frac{△}{\sqrt{2}}\;\frac{\sqrt{\frac{f}{f_{a|d}}}+\sqrt{2}\frac{f}{f_{a|d}}}{1+\sqrt{\frac{2f}{f_{a|d}}}+\frac{f}{f_{a|d}}}}}\;+\;i\underset{ℑ\mu_{\mathrm{Ph}}^{*}}{\underset{︸}{\frac{△}{\sqrt{2}}\;\frac{\sqrt{\frac{f}{f_{a|d}}}}{1+\sqrt{\frac{2f}{f_{a|d}}}+\frac{f}{f_{a|d}}}}}}}]-f^{2}\right\}\times \\ \; & \{f_{0}^{2}[\overset{\mu_{\mathrm{Ch}}^{*}}{\overset{︷}{{\underset{︸}{1+C\sqrt{\frac{f}{2f_{a|d}}}}}_{ℜ\mu_{\mathrm{Ch}}^{*}}+\;\;i\underset{ℑ\mu_{\mathrm{Ch}}^{*}}{\underset{︸}{C\sqrt{\frac{f}{2f_{a|d}}}}}}}+\\ \; & \;\overset{\mu_{\mathrm{Ph}}^{*}}{\overset{︷}{\underset{ℜ\mu_{\mathrm{Ph}}^{*}}{\underset{︸}{\frac{△}{\sqrt{2}}\;\frac{\sqrt{\frac{f}{f_{a|d}}}+\sqrt{2}\frac{f}{f_{a|d}}}{1+\sqrt{\frac{2f}{f_{a|d}}}+\frac{f}{f_{a|d}}}}}\;+\;i\underset{ℑ\mu_{\mathrm{Ph}}^{*}}{\underset{︸}{\frac{△}{\sqrt{2}}\;\frac{\sqrt{\frac{f}{f_{a|d}}}}{1+\sqrt{\frac{2f}{f_{a|d}}}+\frac{f}{f_{a|d}}}}}}}]\left(1-f^{2}\left[1+\chi \right]\right)+f^{2}\left(f^{2}-1\right){\}}^{-1} \end{array}$$

and

(22) $$\begin{array}{cc} \frac{\hat{u}}{U_{\mathrm{st}}}=f^{\alpha }f_{0}^{2} & \left[\overset{\mu_{\mathrm{Ch}}^{*}}{\overset{︷}{\underset{ℜ\mu_{\mathrm{Ch}}^{*}}{\underset{︸}{1+C\sqrt{\frac{f}{2f_{a|d}}}}}+\;\;i\underset{ℑ\mu_{\mathrm{Ch}}^{*}}{\underset{︸}{C\sqrt{\frac{f}{2f_{a|d}}}}}}}+\overset{\mu_{\mathrm{Ph}}^{*}}{\overset{︷}{\underset{ℜ\mu_{\mathrm{Ph}}^{*}}{\underset{︸}{\frac{△}{\sqrt{2}}\;\frac{\sqrt{\frac{f}{f_{a|d}}}+\sqrt{2}\frac{f}{f_{a|d}}}{1+\sqrt{\frac{2f}{f_{a|d}}}+\frac{f}{f_{a|d}}}}}\;+\;i\underset{ℑ\mu_{\mathrm{Ph}}^{*}}{\underset{︸}{\frac{△}{\sqrt{2}}\;\frac{\sqrt{\frac{f}{f_{a|d}}}}{1+\sqrt{\frac{2f}{f_{a|d}}}+\frac{f}{f_{a|d}}}}}}}\right]\times \\ \; & \{f_{0}^{2}[\overset{\mu_{\mathrm{Ch}}^{*}}{\overset{︷}{\underset{ℜ\mu_{\mathrm{Ch}}^{*}}{\underset{︸}{1+C\sqrt{\frac{f}{2f_{a|d}}}}}+\;\;i\underset{ℑ\mu_{\mathrm{Ch}}^{*}}{\underset{︸}{C\sqrt{\frac{f}{2f_{a|d}}}}}}}+\\ \; & \;\overset{\mu_{\mathrm{Ph}}^{*}}{\overset{︷}{\underset{ℜ\mu_{\mathrm{Ph}}^{*}}{\underset{︸}{\frac{△}{\sqrt{2}}\;\frac{\sqrt{\frac{f}{f_{a|d}}}+\sqrt{2}\frac{f}{f_{a|d}}}{1+\sqrt{\frac{2f}{f_{a|d}}}+\frac{f}{f_{a|d}}}}}\;+\;i\underset{ℑ\mu_{\mathrm{Ph}}^{*}}{\underset{︸}{\frac{△}{\sqrt{2}}\;\frac{\sqrt{\frac{f}{f_{a|d}}}}{1+\sqrt{\frac{2f}{f_{a|d}}}+\frac{f}{f_{a|d}}}}}}}]\left(1-f^{2}\left[1+\chi \right]\right)+f^{2}\left(f^{2}-1\right){\}}^{-1}. \end{array}$$

**Figure 7:**
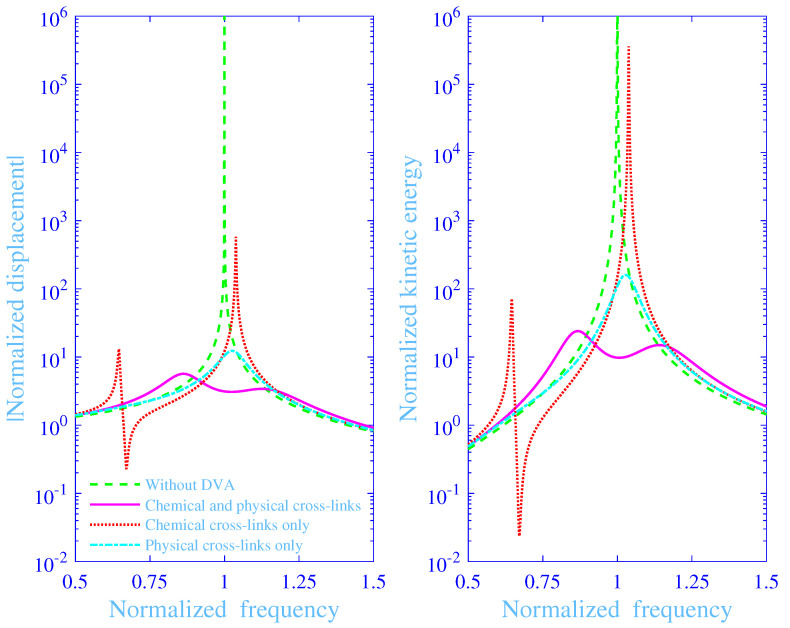
Absolute value of the normalized displacement and the normalized kinetic energy of the the mass *M* versus the normalized frequency with and without the dynamic vibration absorber (DVA) together with the absolute value of the normalized displacement of the mass *m* versus the normalized frequency, with both chemical and physical cross-links, with the chemical cross-links only and with the physical cross-links only, where α=0, χ=1/10 and C=0.04.

The absolute value of the normalized displacement $|\hat{U}/U_{\mathrm{st}}|$ and the normalized kinetic energy $W_{\mathrm{kin}}$ of the mass *M* versus the normalized frequency *f*, with and without the dynamic vibration absorber are in Figure 7, while using the optimized parameter values in Table 2 with α=0, C=0.04 and χ=1/10. The hydrogel dynamic vibration absorber curves with both chemical and physical cross-links are in magenta solid lines (**—**) while the corresponding curves for the chemical cross-links only are in red dotted lines (•••) and for the physical cross-links only in cyan dash–dotted lines (**– •**). The curves without the dynamic vibration absorber are in green dashed lines (**- - -**) in the same Figure 7. Clearly, the curves for the chemical cross-links only display two, sharp resonance peaks—the first located at a normalized frequency well below the normalized natural frequency for the primary vibration system f=1 while the second at a normalized frequency close and just above the same normalized natural frequency. They also show an anti-resonance trough close to the first resonance peak. The peaks are finite and the trough is non-zero due to the exceptionally low, but non-vanishing (C≠0) chemical Rouse type damping with the normalized chemical loss modulus $ℑ\mu_{\mathrm{Ch}}^{*}=C\sqrt{f/2(1+△)}$ and the loss factor $\eta_{\mathrm{Ch}}=1/\left\{1+\sqrt{2(1+△)/f}/C\right\}$—here reading 0.013 and 1.3% at f=1, respectively. The shift of the trough to a lower normalized frequency is due to the substantially lower absolute value of the normalized shear modulus for the chemical cross-links only compared to the results with both the chemical and physical cross-links. Here, the ratio $|\mu_{\mathrm{Ch}}/\mu_{\mathrm{Ch}+\mathrm{Ph}}|\approx 46\%$ at f=1, thus the absolute value of the normalized chemical modulus is less than half of the corresponding normalized total modulus with both the chemical and physical cross-links active at f=1. Furthermore, the curves for the physical cross-links only clearly display a broad, well-damped resonance peak at a normalized frequency close and just above the normalized natural frequency for the primary vibration system f=1. The high damping is not surprising as the hydrogel network with the physical cross-links only displays a high adhesion–deadhesion activity of the cross-links with an exceptionally high normalized physical loss modulus $ℑ\mu_{\mathrm{Ph}}^{*}=△/\sqrt{2}\left\{\sqrt{(1+△)/f}+\sqrt{2}+\sqrt{f/(1+△)}\right\}$ and an exceptionally loss factor $\eta_{\mathrm{Ph}}=1/\left\{1+\sqrt{2f/(1+△)}\right\}$—here reading 0.66 and 61% at f=1, respectively. Finally, the inclusion of both the chemical and physical cross-links significantly reduces the absolute value of the normalized displacement and the normalized kinetic energy of the mass *M*, displaying a smooth normalized frequency dependence over the normalized frequency region considered in Figure 7 with the normalized (total) loss modulus $ℑ\mu_{\mathrm{Ch}+\mathrm{Ph}}^{*}\approx 0.67$ and the loss factor $\eta_{\mathrm{Ch}+\mathrm{Ph}}\approx 32\%$ at f=1, thus displaying a more moderate loss factor as compared to the case with the physical cross-links only.

## 4. Conclusions

The recent material developing progress into tough hydrogels opens up for alternative applications beyond their normal usage in tissue engineering. In this paper it is theoretically concluded that doubly cross-linked, single polymer network hydrogels containing both chemical and physical cross-links are a plausible material in dynamic vibration absorber springs. They show a broad frequency spectrum with a high loss factor due to the intensive adhesion–deadhesion activities of the physical cross-links resulting in a significant vibration reduction and a smooth frequency dependence of the primary vibration system. An interesting continuation of the work performed is to practically test a suitable single polymer network hydrogel that follows the stress–strain relation applied in this study, in addition to investigate other mechanical properties including its fatigue, ageing and wear resistance. Those aspects involve a great deal of work and are beyond the scope of the present paper. The results of the study in the paper are useful in designing dynamic vibration absorbers made of this interesting tough polymer hydrogel in order to reduce for example aircraft and spacecraft vibrations.

## Figures and Tables

**Table 1:** Optimized hydrogel dynamic vibration damping properties in green at C=0 with $f_{a|d}\;=\;1\;+\;△$, $f_{-}=0.5$ and $f_{+}=1.5$.

α	−2			0			+2		
χ	$f_{0}$	△	$f_{a|d}$	$f_{0}$	△	$f_{a|d}$	$f_{0}$	△	$f_{a|d}$
1/5	0.5139	8.3062	9.3062	0.5527	9.1548	10.1548	0.5760	13.1228	14.1228
1/10	0.6408	3.7198	4.7198	0.6661	3.9055	4.9055	0.6630	5.2817	6.2817
1/20	0.7396	1.9690	2.9690	0.7554	2.0106	3.0106	0.7594	2.2679	3.2679
1/40	0.8133	1.1520	2.1520	0.8224	1.1625	2.1625	0.8273	1.2207	2.2207

**Table 2:** Optimized hydrogel dynamic vibration damping properties in green at C=0.04 with $f_{a|d}=1+△$, $f_{-}=0.5$ and $f_{+}=1.5$.

α	−2			0			+2		
χ	$f_{0}$	△	$f_{a|d}$	$f_{0}$	△	$f_{a|d}$	$f_{0}$	△	$f_{a|d}$
1/5	0.5141	8.2145	9.2145	0.5529	9.0587	10.0587	0.5762	13.0316	14.0316
1/10	0.6418	3.6178	4.6178	0.6672	3.8005	4.8005	0.6639	5.1755	6.1755
1/20	0.7423	1.8576	2.8576	0.7582	1.8975	2.8975	0.7620	2.1538	3.1538
1/40	0.8184	1.0325	2.0325	0.8277	1.0422	2.0422	0.8325	1.0998	2.0998

**Table 3:** Optimized hydrogel and natural rubber dynamic vibration damping properties in green at various natural frequencies for the primary vibration system $\Omega_{0}$ with α=0, χ=1/10, $f_{-}=0.5$ and $f_{+}=1.5$.

Material	$f_{0}$	△	$f_{a|d}$	C	Exponent Equation (9)	$\min[{\bar{W}}_{\mathrm{kin}}]$ Equation (20)
Hydrogel from Table 2	0.6672	3.8005	4.8005	0.04	${\left(if/f_{a|d}\right)}^{0.5}$	8.1488
Hydrogel full optimization	0.7336	4.7051	$1.9434\;\times \;10^{1}$	0.04	${\left(if/f_{a|d}\right)}^{0.5}$	8.1372
NR $\Omega_{0}=1\;$rad/s	0.9773	276	$3.3987\;\times \;10^{8}$	0	${\left(if/f_{a|d}\right)}^{0.657}$	$2.6093\;\times \;10^{3}$
NR $\Omega_{0}=10\;$rad/s	0.9665	276	$3.3987\;\times \;10^{7}$	0	${\left(if/f_{a|d}\right)}^{0.657}$	$5.7875\;\times \;10^{2}$
NR $\Omega_{0}=100\;$rad/s	0.9333	276	$3.3987\;\times \;10^{6}$	0	${\left(if/f_{a|d}\right)}^{0.657}$	$1.3763\;\times \;10^{2}$
